# diTFPP, a Phenoxyphenol, Sensitizes Hepatocellular Carcinoma Cells to C_2_-Ceramide-Induced Autophagic Stress by Increasing Oxidative Stress and ER Stress Accompanied by LAMP2 Hypoglycosylation

**DOI:** 10.3390/cancers14102528

**Published:** 2022-05-20

**Authors:** Chien-Chih Chiu, Yen-Chun Chen, Yung-Ding Bow, Jeff Yi-Fu Chen, Wangta Liu, Jau-Ling Huang, En-De Shu, Yen-Ni Teng, Chang-Yi Wu, Wen-Tsan Chang

**Affiliations:** 1 Department of Biotechnology, Kaohsiung Medical University, Kaohsiung 807, Taiwan; cchiu@kmu.edu.tw (C.-C.C.); r020135@gap.kmu.edu.tw (Y.-C.C.); yifuc@kmu.edu.tw (J.Y.-F.C.); liuwangta@kmu.edu.tw (W.L.); u110554003@gap.kmu.edu.tw (E.-D.S.); cywu@mail.nsysu.edu.tw (C.-Y.W.); 2 Department of Biological Sciences, National Sun Yat-sen University, Kaohsiung 804, Taiwan; 3 The Graduate Institute of Medicine, Kaohsiung Medical University, Kaohsiung 807, Taiwan; 4 Department of Medical Research, Kaohsiung Medical University Hospital, Kaohsiung 807, Taiwan; 5 Center for Cancer Research, Kaohsiung Medical University, Kaohsiung 807, Taiwan; 6 Ph.D. Program in Life Sciences, College of Life Science, Kaohsiung Medical University, Kaohsiung 807, Taiwan; u109850001@kmu.edu.tw; 7 Department of Bioscience Technology, College of Health Science, Chang Jung Christian University, Tainan 711, Taiwan; jaulingh@mail.cjcu.edu.tw; 8 Department of Biological Sciences and Technology, National University of Tainan, Tainan 700, Taiwan; tengyenni@mail.nutn.edu.tw; 9 Division of General and Digestive Surgery, Department of Surgery, Kaohsiung Medical University Hospital, Kaohsiung 807, Taiwan; 10 Department of Surgery, School of Medicine, College of Medicine, Kaohsiung Medical University, Kaohsiung 807, Taiwan

**Keywords:** hepatocellular carcinoma (HCC), ceramide, sphingolipid metabolism, phenoxyphenol compound, diTFPP, oxidative stress, autophagic stress, endoplasmic reticulum (ER) stress, LAMP2 glycosylation

## Abstract

**Simple Summary:**

Chemotherapy is the major treatment modality for advanced or unresectable hepatocellular carcinoma (HCC). Unfortunately, chemoresistance carries a poor prognosis in HCC patients. Exogenous ceramide, a sphingolipid, has been well documented to exert anticancer effects; however, recent reports showed ceramide resistance, which limits the development of the ceramide-based cancer treatment diTFPP, a novel phenoxyphenol compound that has been shown to sensitize HCC cells to ceramide treatment. Here, we further clarified the mechanism underlying diTFPP-mediated sensitization of HCC to C_2_-ceramide-induced stresses, including oxidative stress, ER stress, and autophagic stress, especially the modulation of LAMP2 glycosylation, the lysosomal membrane protein that is crucial for autophagic fusion. This study may shed light on the mechanism of ceramide resistance and help in the development of adjuvants for ceramide-based cancer therapeutics.

**Abstract:**

Hepatocellular carcinoma (HCC), the most common type of liver cancer, is the leading cause of cancer-related mortality worldwide. Chemotherapy is the major treatment modality for advanced or unresectable HCC; unfortunately, chemoresistance results in a poor prognosis for HCC patients. Exogenous ceramide, a sphingolipid, has been well documented to exert anticancer effects. However, recent reports suggest that sphingolipid metabolism in ceramide-resistant cancer cells favors the conversion of exogenous ceramides to prosurvival sphingolipids, conferring ceramide resistance to cancer cells. However, the mechanism underlying ceramide resistance remains unclear. We previously demonstrated that diTFPP, a novel phenoxyphenol compound, enhances the anti-HCC effect of C_2_-ceramide. Here, we further clarified that treatment with C_2_-ceramide alone increases the protein level of CERS2, which modulates sphingolipid metabolism to favor the conversion of C_2_-ceramide to prosurvival sphingolipids in HCC cells, thus activating the unfolded protein response (UPR), which further initiates autophagy and the reversible senescence-like phenotype (SLP), ultimately contributing to C_2_-ceramide resistance in these cells. However, cotreatment with diTFPP and ceramide downregulated the protein level of CERS2 and increased oxidative and endoplasmic reticulum (ER) stress. Furthermore, insufficient LAMP2 glycosylation induced by diTFPP/ceramide cotreatment may cause the failure of autophagosome–lysosome fusion, eventually lowering the threshold for triggering cell death in response to C_2_-ceramide. Our study may shed light on the mechanism of ceramide resistance and help in the development of adjuvants for ceramide-based cancer therapeutics.

## 1. Introduction

Liver cancer is the leading cause of cancer-associated death worldwide, including in Taiwan [1,2]. Hepatocellular carcinoma (HCC) originates from hepatocytes and accounts for 85% of all primary liver cancer cases [3]. Despite novel developments in HCC treatments, advanced HCC often develops chemoresistance [4,5,6]. Generally, agents kill and inhibit the growth of HCC cells via mechanisms associated with apoptosis induction. Nevertheless, cancer cells finally escape apoptotic signaling and become more chemoresistant, resulting in chemotherapeutic failure. Similarly, acquired resistance to anticancer drugs is the hallmark of HCC progression. Therefore, chemotherapeutic applications against HCC seem to have plateaued [7,8,9,10]. Cancer cells acquire chemoresistance via many mechanisms, including enhancing drug efflux and sequestration, inhibiting drug uptake, or inactivating drug metabolism [11].

Ceramide is a sphingolipid and is the central molecule of sphingolipid metabolism that can be synthesized into many sphingolipids [12]. Ceramide-based compounds are widely used to treat cancers [13,14,15,16], including HCC [17]. Short-chain ceramides, such as C_2_-, C_6_-, and C_8_-ceramide, prevent cancer proliferation and invasion [14,18,19,20]. Furthermore, a dramatic increase in endogenous oxidative stress was associated with ceramide treatment [14]. Ceramides have been shown to exert direct cytotoxicity against various cancers [14,20]. Despite the abovementioned advantages of ceramide, side effects, including acquired drug resistance, are frequently observed with ceramide treatment [19]. Moreover, HCC cells themselves are usually chemoresistant, and the response rate of HCC to chemotherapy is only approximately 10–20%, resulting in poor overall survival for patients with advanced HCC [21,22]. Cotreatment with ceramide and drugs enhances cytotoxicity in cancer cells, such as lung [13,23] and liver cancer cells [17,24].

ER stress is a physiological process that cells use to precisely regulate intracellular protein quality and activate an adaptive mechanism called the unfolded protein response (UPR). During the UPR, three pathways—IRE1α, PERK, and ATF6—are activated; these pathways activate the transcription of UPR target genes to respond to endoplasmic reticulum (ER) stress [25]. Unfortunately, the UPR is also a target for cancer progression; previous evidence indicated that UPR activation in cancer cells can lead to favorable conditions, such as cell survival, dormancy, immunosuppression, and metastasis, for these cells [26]. Ceramide accumulation was found to induce ER stress. In a previous study, exogenous ceramide treatment was reported to disrupt ER calcium homeostasis, resulting in ER stress via PERK-mediated eIF2α activation and apoptosis [27]. In nonalcoholic fatty liver disease, ceramide synthases are considered to regulate ER stress-mediated triglyceride accumulation [28]. Our previous study showed that phenoxyphenol derivatives exert anticancer effects in HCC [24,29] and non-small-cell lung cancer cells [30,31] by modulating reactive oxygen species (ROS) production. Furthermore, ROS modulation was suggested to be a promising strategy for inducing apoptosis in cancer cells by increasing ER stress [32,33].

Protein degradation processes occurring during ER stress include lysosome-mediated protein degradation via autophagy and proteasome-mediated degradation [34]. During the UPR, cleaved ATF6 and XBP1 increase the expression of genes implicated in the endoplasmic reticulum-associated protein degradation (ERAD) machinery [35]. The ERAD machinery then recognizes and removes misfolded proteins from the ER, after which the proteins are exported to the cytoplasm, where they are degraded by the ubiquitin–proteasome pathway [36]. When ERAD fails to remove misfolded proteins adequately, autophagy is activated as an alternate route to regulate excess proteins [36].

Autophagy is a critical degradation system regulated by the lysosome by which unfolded/misfolded proteins, protein aggregates, and damaged organelles are recycled and eliminated from cells. However, it was identified as an important protective mechanism under ER stress conditions [37]. First, targets are sequestered into autophagosomes via autophagy. The autophagosome then fuses with a lysosome, and lysosomal hydrolases further degrade the contents [34].

Autophagy is critical to the balance between cell survival and death. Cells tend to survive when adaptive autophagy is activated. However, improper activation of autophagy can cause lysosomal dysfunction and increase autophagic stress, resulting in cell death. Lysosome-associated membrane protein 2 (LAMP2) is a major lysosomal membrane protein with a molecular mass of 45 kDa. The extensive glycosylation of LAMP2 by adding various N- and O-linked oligosaccharides (with molecular masses of approximately 100–110 kDa) is essential for mature and functional lysosomes. Lysosomal dysfunction may cause an imbalance in autophagy, resulting in increased autophagic stress and eventual cytotoxicity in cells [38]. LAMP2 interacts with Golgi reassembly stacking protein of 55 kDa (GRASP55), which is de-O-GlcNAcylated under glucose deprivation, and GRASP55 interacts with LC3-II, which acts as a membrane tether to enable autophagosome–lysosome fusion [39]. The major post-translational modifications of LAMP2 include proteolytic cleavage [40] and glycosylation, which affect variations in its molecular mass, and hyperglycosylation of LAMPs protects the lysosomal membrane from self-digestion [41]. In contrast, de- or hypo-glycosylation of LAMP2 inhibits autophagosome–lysosome fusion, causing an increase in autophagic stress in cells.

Phenoxyphenol derivatives have been shown to inhibit cancer cells, including colon and prostate cancer cells [42,43]. Parsai et al. reported that a panel of phenoxyphenol derivatives exhibited anti-inflammatory and antimetastatic activities by binding to the active sites of matrix metalloproteinase 9 (MMP-9) and cyclooxygenase 2 (COX-2) [44]. Recently, we identified phenoxyphenol 4-[4-(4-hydroxyphenoxy)phenoxy]phenol (4-HPPP), which selectively inhibits HCC cells [29] and lung cancer cells through the accumulation of γH2AX, a DNA damage marker; impairs autophagy; and finally, induces apoptosis [31]. The 4-HPPP derivative diTFPP modulates ceramide metabolism to inhibit the transformation of proapoptotic ceramide to antiapoptotic ceramide, resulting in apoptotic cell death in HCC cells stimulated with exogenous C_2_-ceramide [24]. The results also indicated autophagy activation and ROS accumulation during diTFPP/C_2_-ceramide treatment [24]. Although we previously demonstrated the effect of diTFPP on enhancing C_2_-ceramide-induced proliferation inhibition in HCC cells, the molecular mechanism underlying diTFPP-mediated HCC sensitization by C_2_-ceramide treatment needs to be elucidated.

This study further examined whether diTFPP treatment increases C_2_-ceramide-induced cytotoxicity and cell death in HCC cells by modulating stresses, especially oxidative stress, ER stress, and autophagic stress. Furthermore, the mechanism underlying diTFPP-mediated sensitization of HCC cells to C_2_-ceramide is proposed in the study.

## 2. Materials and Methods

### 2.1. Cell Culture

The Bioresource Collection and Research Center (BCRC; Hsinchu, Taiwan) provided HA22T/VGH (HA22T, #60168) and HA59T/VGH (HA59T, #60169) cells, which were maintained in a 3:2 mixture of Dulbecco’s modified Eagle’s medium and Ham’s F-12 Nutrient Mixture (DMEM/F12, 3:2; HIMEDIA, Mumbai, India) supplemented with 8% fetal bovine serum. Cells were cultured in a 37 °C incubator with 5% CO_2_.

### 2.2. Assessment of Cell Viability

A trypan blue exclusion assay was used to determine cell viability [24]. Briefly, in a 12-well plate, 2 × 10^4^ cells were seeded per well. After overnight incubation, these cells were resuspended with trypsin (#TCL034, HIMEDIA, Mumbai, India), stained with trypan blue, and counted and evaluated for viable cells negative for trypan blue staining.

### 2.3. Colony Formation Assay

A total of 400 cells were plated overnight in 12-well plates. For 72 h, these cells were cotreated with diTFPP and C_2_-ceramide. The medium was then replenished, and the cells were grown for 7 days. These cells were fixed in 4% paraformaldehyde, and subsequently washed and stained with 0.1% Giemsa (Cat. No. 1.09204.1000, Merck-Millipore Ltd., Tullagreen, Carrigtwohill, County Cork, Ireland).

### 2.4. Western Blotting

Cells were seeded at a density of 5 × 10^5^ cells per 10 cm dish overnight. Cells were harvested and lysed with 100 μL of 1 × RIPA buffer (#20-188, Merck Millipore Ltd.), and the protein concentrations in the lysate were determined using a BCA protein kit (Pierce, Rockford, IL, USA). Proteins were separated by sodium dodecyl sulfate–polyacrylamide gel electrophoresis (SDS–PAGE) and electrotransferred to polyvinylidene fluoride (PVDF) membranes (Merck Millipore Ltd.). A prestained protein marker (#TM-PM10170, Tools, New Taipei City, Taiwan) was used. The PVDF membranes were blocked with 5% nonfat milk in TBS buffer with 0.1% Tween 20 for 1 h and were then incubated with primary antibodies against ATF6 (1:3000, #A12570, ABclonal, Woburn, MA, USA), BIP (1:1000, #A11366, ABclonal), CATHEPSIN B (1:1000, #ab125067, Abcam, Cambridge, UK), CATHEPSIN D (1:1000, #A19680, ABclonal), CERS2 (1:1000, #A18236, ABclonal), CHOP (1:2000, #15204-1-AP, Proteintech, Wuhan, Hubei, China), IRE1α (1:2000, #3294, Cell Signaling Technology, Danvers, MA, USA), p-IRE1α-S724 (1:2000, #AP0878, ABclonal), LAMP2 (1:1000, #ab125068, Abcam), LC3B (1:3000, #2775s, Cell Signaling Technology), PERK (1:2000, #A18196, ABclonal), and the internal controls β-actin (1:5000, #sc-47778, Santa Cruz Biotechnology, Dallas, TX, USA) α-tubulin (1:5000, #sc-5286, Santa Cruz Biotechnology), and GAPDH (1:8000, #MAB374, Merck Millipore Ltd.). The PVDF membranes were further incubated with the corresponding horseradish peroxidase (HRP)-conjugated secondary antibodies (1:10,000, #20102 for goat anti-mouse IgG, Leadgene Biomedical Inc., Tainan, Taiwan; 1:10,000, #7074, Cell Signaling Technology). Then, the signals of specific proteins were detected with an enhanced chemiluminescence (ECL) detection kit (PerkinElmer Inc., Waltham, MA, USA). The Western blots are shown in Supplementary File S1 in their entirety.

### 2.5. Flow Cytometric Detection of ROS and Lysosomes

Cells were seeded at a density of 5 × 10^4^ cells per well in a 6-well plate overnight. The cells were treated with the indicated concentrations of diTFPP (from 5 to 10 M) and 20 μM C_2_-ceramide for 24 h. The treated cells were stained at 37 °C with 0.1 μM dihydroethidium (DHE; D11347, Thermo Fisher Scientific, Waltham, MA, USA) or 50 nM LysoTracker™ Red DND-99 (#L7528, Thermo Fisher Scientific). The cells were washed in phosphate-buffered saline (PBS) to remove dye and were analyzed on an LSR II flow cytometer with 488-nm bandpass blue excitation filters and 590-nm (red) barrier filters provided by Kaohsiung Medical University, Taiwan.

### 2.6. Next-Generation Sequencing (NGS) Analysis

*RNA-seq* for gene expression profiling was performed by a company (BIOTOOLS, Taipei, Taiwan) [24]. In brief, the cDNA molecules were synthesized, purified, terminal repaired, A-tailed, sequence adaptor-ligated, and enriched by PCR. Sequencing. The sequencing data were subjected to clustering analysis using the Expander 7 software [45], and gene ontology (GO) analysis was performed using the Database for Annotation, Visualization, and Integrated Discovery (DAVID) website (version 6.8) [46]. The ClustVis website [47] was used to generate the principal component analysis (PCA) plot and heatmap. The biological process differences in the C_2_-ceramide and C_2_-ceramide/diTFPP groups were investigated using gene set enrichment analysis (GSEA). The gene set was created using the following thresholds: false discovery rate (FDR) < 0.25 and *p* < 0.05.

### 2.7. LC3 Turnover Assay

Assessment of LC3 turnover is based on the degradation of LC3-II in autolysosomes, which is a widely used approach for measuring autophagic flux [48]. HA22T cells were seeded at a density of 5 × 10^5^ cells per 10 cm dish overnight. Cells were cotreated with diTFPP/C_2_-ceramide in the presence and absence of 100 μM chloroquine (CQ; an inhibitor that blocks autophagosome–lysosome fusion, Sigma Aldrich) for 24 h and were then analyzed by Western blotting with anti-LC3 antibodies. The relative amount of LC3-II (the relative fold changes) between the control and diTFPP/ceramide groups in the presence or absence of chloroquine, an inhibitor of late-stage autophagy, and the formulae used to calculate formation, degradation, and net turnover [49].

### 2.8. Assessment of Lipofuscin Clearance

Cells were seeded at a density of 1 × 10^4^ cells per well in a 24-well plate overnight. The cells were treated with the indicated concentrations of diTFPP (from 5 to 10 M) and 20 μM C_2_-ceramide for 24 h. Cells were fixed with 4% paraformaldehyde, and lipofuscin was stained with 0.1% Sudan Black B (SBB; in 70% ethanol as the solvent). The cells were then washed with 70% ethanol to remove excess dye. Nuclei were stained with 0.1% nuclear fast red. The stained cells were observed and counted under a microscope (IX71, Olympus Corporation, Tokyo, Japan)

### 2.9. Senescence-Associated β-galactosidase (SA-gal) Staining

An SA-gal staining assay was performed to evaluate cellular senescence [47]. The protocol utilized in the study was based on a previously published protocol [16] with minor modifications. In summary, cells were seeded at a density of 1 × 10^4^ cells per well in a 24-well plate overnight, cotreated for 24 h with diTFPP/C_2_-ceramide, washed with PBS, fixed with glutaraldehyde, and incubated with 5-bromo-4-chloro-3-indolyl β-galactoside (X-gal) for 24 h (X-gal was dissolved in dimethylformamide with 5 mM potassium ferrocyanide, 150 mM NaCl, 40 mM citric acid, 40 mM sodium phosphate, and 2 mM MgCl_2_ (pH 6.0)) for 24 h. Then, the stained cells were washed with PBS, and the green-stained cells were considered senescent cells.

### 2.10. Detection of Lysosomes and Lysosomal pH Using Fluorescence Microscopy

The procedures for the detection of lysosomes and changes in lysosomal pH were as described in a previous study [50]. In brief, HCC cells were seeded at a density of 1 × 10^4^ cells per well in a 24-well plate overnight and cotreated for 24 h with diTFPP/C_2_-ceramide stained with 50 nM LysoTracker™ Red DND-99 (Thermo Fisher Scientific) or 1 μM LysoSensor™ Yellow/Blue DND-160 (#L7545, Thermo Fisher Scientific) for 20 min at 37 °C. Then, the cells were washed with PBS and observed by fluorescence microscopy (IX71, Olympus).

### 2.11. Immunofluorescence Analysis

Immunofluorescence staining was used to examine the subcellular localization of specific proteins, including CATHEPSIN B (1:500, #ab125067, Abcam) and LAMP2 (1:500, #sc-18822, Santa Cruz Biotechnology). HCC cells were seeded at a density of 1 × 10^4^ cells per well in a 24-well plate overnight and cotreated for 24 h with diTFPP/C_2_-ceramide. diTFPP/C2-ceramide-treated cells were incubated first with primary antibodies against the specific proteins and then with rabbit IgG-iFluor 594 (1:500, #C04031, Croyez Bioscience Co., excitation 590 nm/emission 610 nm) or mouse IgG-FITC (1:500, #GTX26816, GeneTex, International Corporation, Hsinchu, Taiwan) secondary antibodies. The nuclear fluorescent dye 4’,6-diamidino-2-phenylindole (DAPI) was used, and images of the cells were acquired with an epifluorescence microscope.

### 2.12. Statistical Analysis

One-way analysis of variance (ANOVA) was used to compare two separate groups, and Student’s *t* test was used for pairwise comparisons. Statistical significance was defined as a *p* value < 0.05.

## 3. Results and Discussion

### 3.1. diTFPP Sensitizes HCC Cells to C_2_-Ceramide-Induced Cytotoxicity and Suppression of Clonogenicity

Phenoxyphenol derivatives have been reported to possess efficacy in the prevention and treatment of cancer, including colon and prostate cancer [42,43]. Our laboratory previously identified a phenoxyphenol-based compound, 4-[2,3,5,6-tetrafluoro-4-(4-hydroxyphenoxy)phenoxy]phenol (TFPP), that significantly enhances camptothecin-induced apoptosis in lung cancer cells by modulating endogenous ROS and ERK signaling [30]. In addition, we demonstrated that another phenoxyphenol compound, 4-HPPP, selectively kills HCC [29] and lung cancer cells [31]. More recently, we showed that the phenoxyphenol diTFPP enhanced the anti-HCC effect of C_2_-ceramide by modulating ceramide metabolism [24].

Given the potential adjuvant effect of diTFPP on C_2_-ceramide-induced cytotoxicity in HCC cells, we further investigated diTFPP to better understand its modulation of stresses, especially oxidative stress, ER stress, and autophagic stress, which are frequently overactivated in cancer cells.

**Figure 1:**
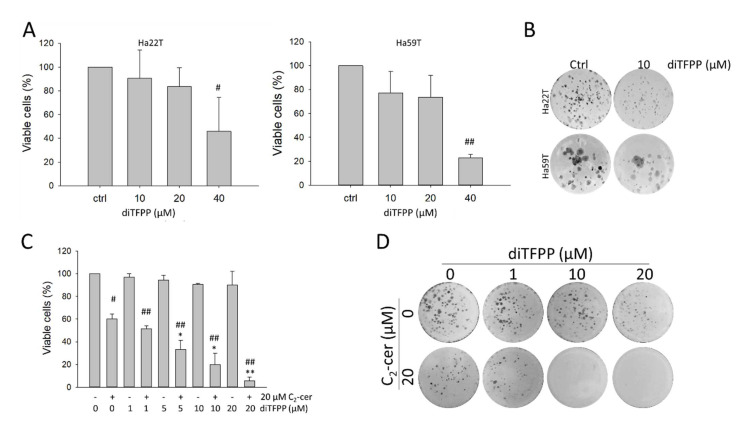
Effect of diTFPP/C_2_-ceramide cotreatment on the proliferation and viability of HCC cells. (**A**) The viability of HCC cells following diTFPP treatment was assessed using the trypan blue exclusion assay for 24 h. (**B**) The effect of diTFPP on the long-term clonogenicity of HCC cells was assessed after diTFPP treatment for 7 days. (**C**) The viability of HA22T cells was assessed after treatment with diTFPP alone or cotreated with C_2_-ceramide for 24 h. (**D**) Colony formation of HA22T cells was assessed after C_2_-ceramide and/or diTFPP treatment for 7 days. # *p* < 0.05 and ## *p* < 0.01; the control group compared with the indicated treatment groups. * *p* < 0.05 and ** *p* < 0.01; the C_2_-ceramide alone group compared with the indicated treatment groups. All data are presented as the mean ± SD of three independent assays.

Figure 1A shows that, at concentrations of less than 20 μM, diTFPP did not produce substantial cytotoxicity in the HCC cell lines HA22T and HA59T. Additionally, the results of the colony formation assay revealed that 10 μM diTFPP considerably reduced the clonogenicity of HCC cells (Figure 1B), indicating the low cytotoxicity of diTFPP in HCC cells.

Our results showed that HA22T cells were resistant to C_2_-ceramide, which led to a cell death rate of less than 40% at a concentration of 20 μM. Under cotreatment with diTFPP and 20 μM C_2_-ceramide, the cell death rate increased to 70%, and diTFPP alone did not exhibit cytotoxicity in HA22T cells (Figure 1C). We performed a colony formation assay to assess whether diTFPP affects the long-term clonogenicity of HCC cells, and the results indicated that diTFPP significantly inhibited colony formation during C_2_-ceramide treatment (Figure 1D), suggesting that diTFPP disrupts endogenous ceramide metabolism, consistent with our previously published hypothesis [24] (Figure S1).

### 3.2. diTFPP Alone Sufficiently Induces ROS Production in HCC Cells

**Figure 2:**
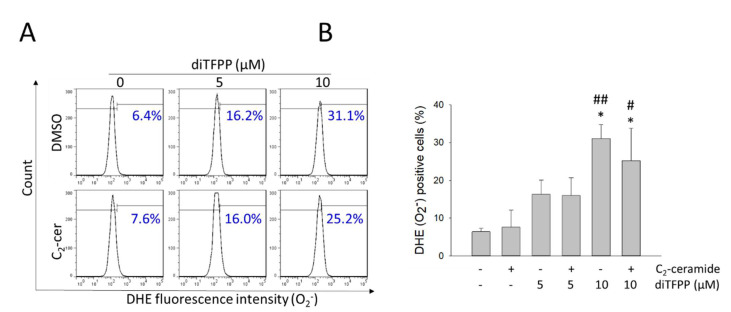
Effect of diTFPP on C_2_-ceramide-induced endogenous ROS production in HCC cells. (**A**) Endogenous ROS were evaluated in cells treated with 20 μM C_2_-ceramide alone and in combination with diTFPP for 24 h using a DHE fluorescence-based flow cytometric assay. (**B**) Quantitative analysis of the data in (**A**). # *p* < 0.05 and ## *p* < 0.01; the control group compared with the indicated treatment groups. * *p* < 0.05; the group treated with C_2_-ceramide alone compared with the indicated treatment groups. All data are presented as the mean ± SD of three independent assays.

ROS are intracellular free radical or nonradical oxygen species, such as superoxide anion and hydrogen peroxide, and dysregulation of ROS production may cause oxidative stress and is highly correlated with various diseases, such as cancer, inflammatory diseases, and vascular diseases [51]. Moderate (nanomolar) levels of ROS can be found in cancer cells and promote tumor initiation, progression, and metastasis [51,52]. However, a high (micromolar) level of endogenous ROS acts as a double-edged sword and can increase susceptibility to ROS-induced apoptotic cell death [51,53]. Thus, modulation of intracellular ROS production could be a promising therapeutic strategy for cancer [54]. Additionally, PJ-34, a PARP inhibitor, upregulates the expression of NADPH oxidases-1 and -4, resulting in ROS production in the ovarian cancer cell lines A2780 and HO8910 [55]. To better understand the mechanism by which diTFPP sensitizes HCC cells to exogenous ceramide treatment, we first assessed the changes in endogenous ROS by flow cytometry-based DHE staining. Treatment with diTFPP alone significantly increased O_2_^−^ production, regardless of combination with C_2_-ceramide (Figure 2), suggesting that diTFPP increases oxidative stress by increasing endogenous ROS production and sensitizing HCC cells to ceramide-induced cell death.

### 3.3. diTFPP Enhances C_2_-Ceramide-Induced ER Stress in HCC Cells

**Figure 3:**
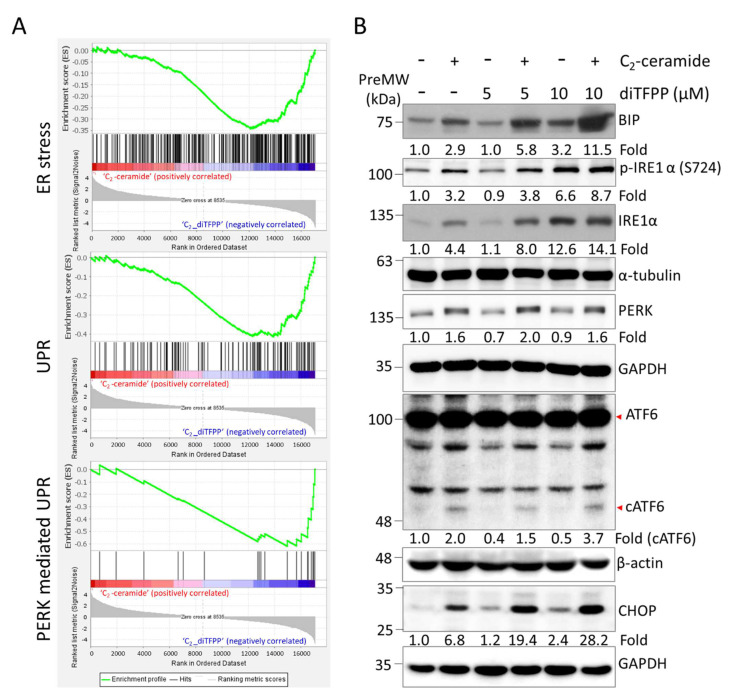
diTFPP mediated ER stress upon cotreatment with 20 μM C_2_-ceramide for 24 h. (**A**) GSEA of C_2_-ceramide-treated cells compared with diTFPP/C_2_-ceramide-treated cells indicated that ER stress response genes were upregulated in the diTFPP/C_2_-ceramide treatment group. (**B**) Western blot analysis of three major ER stress pathway markers. PreMW: prestained protein molecular weight marker (see Section 2.4).

Increased production of ROS is usually associated with the accumulation of unfolded or misfolded proteins, which has been reported to be highly correlated with ER stress. For example, protodioscin, a steroidal saponin, was reported to initiate ER stress-induced apoptosis in human cervical cancer cells by modulating ROS production [32]. Similarly, plumbagin, a naphthoquinone isolated from *Plumbago zeylanica*, was shown to increase intracellular ROS and, consequently, induce ER stress-mediated apoptosis in prostate cancer cells [33]. To further investigate the potential mechanism of diTFPP-induced sensitization in ceramide-induced HCC cells, we first performed NGS-based GSEA of cells subjected to C_2_-ceramide and C_2_-ceramide/diTFPP treatment (Figure 3A). The NGS analysis revealed that, as compared with treatment with C_2_-ceramide alone, cotreatment with diTFPP and C_2_-ceramide induced the expression of ER stress-associated genes, suggesting that diTFPP/C_2_-ceramide treatment enhanced the UPR. An ER chaperone, BIP, is considered a marker of ER stress and is overexpressed when misfolded proteins accumulate and activate downstream ER stress pathways, including the IRE1α, PERK, and ATF6 pathways [56]. In this study, the Western blot analysis results indicated that BIP accumulated in the diTFPP/C_2_-ceramide-treated groups, indicating ER stress induction (Figure 3B). The unfolded protein response (UPR) is essential for preventing the accumulation of unfolded or misfolded proteins and maintaining ER homeostasis. On the other hand, excessive amounts of misfolded proteins are generated by starvation and anticancer drug-induced mitochondrial malfunction. In contrast, ER stress means that excess misfolded proteins caused by starvation and anticancer drug-induced mitochondrial dysfunction can increase ROS accumulation and cell death [57]. BIP binds to three transmembrane proteins in the ER lumen, including IRE-1 (inositol-requiring transmembrane kinase/endoribonuclease), PERK (protein kinase RNA-activating (PKR)-like ER kinase), and ATF6 (activating transcription factor 6) [56,58], preventing their activation and downstream UPR signaling. Activated ATF4 also promotes the expression of CHOP, an apoptotic Bim transcription factor that inhibits pro-apoptotic proteins such as Bcl-2, Bcl-xL, and Mcl-1 [59,60].

Similarly, our study indicated that all three canonical ER stress pathways, IRE1α, PERK, and ATF6, along with the downstream CHOP pathway, were activated during diTFPP/C_2_-ceramide treatment (Figure 3B).

### 3.4. diTFPP Enhances C_2_-Ceramide-Induced Autophagic Stress in HCC Cells

Zhang et al. used the term “autophagic flux” to describe the whole autophagic process, including autophagosome formation, maturation, fusion with lysosomes, and the degradation of autophagic substrates inside lysosomes. Autophagic flux assays are critical for assessing the dynamic autophagy process because they help to distinguish between autophagosome accumulation owing to increased autophagic activity and autophagosome accumulation due to poor lysosomal clearance [61]. Furthermore, Ainhoa Plaza-Zabala et al. suggested that the LC3 turnover assay analyzes not only autophagic flux but also autophagosome formation and degradation [49].

**Figure 4:**
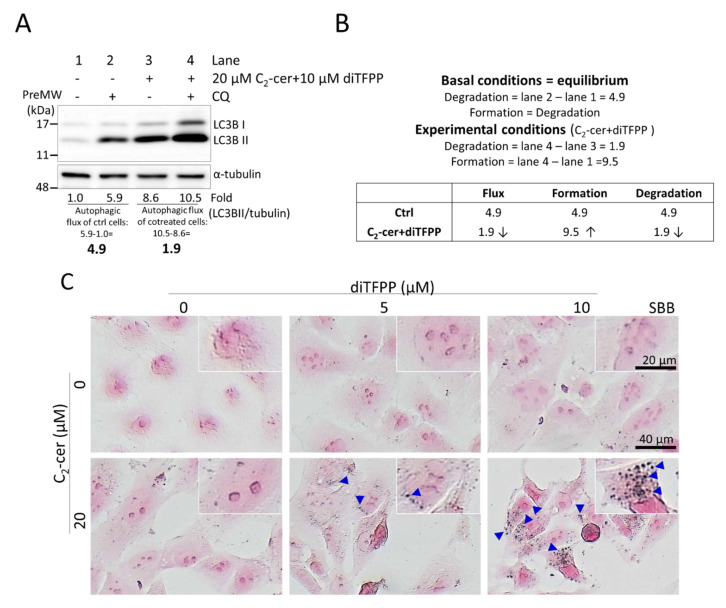

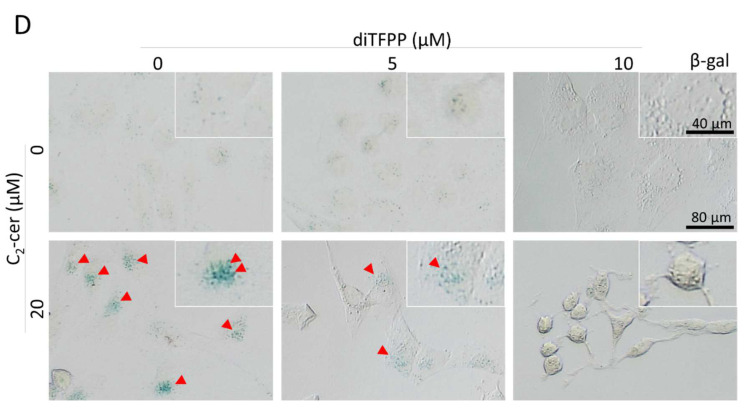
Assessment of autophagic flux after diTFPP/C_2_-ceramide treatment for 24 h. (**A**) Autophagic flux was assessed by measuring the level of LC3B II protein remaining after CQ treatment (see Section 3.4). (**B**) Quantitative results of experiments (with and without C_2_-ceramide/diTFPP) in the presence or absence of an autophagy inhibitor (CQ) were calculated to assess the formation and degradation of autophagosomes using the formula which was described by Plaza-Zabala, A. et al. [49]. Assessment of lipofuscin accumulation using SBB staining (**C**) and senescence using SA-gal staining (**D**). The blue arrows indicate lipofuscin, and the red arrows indicate cells positive for SA-gal staining, the hallmark of senescence.

Accumulation of lipofuscin, a protein–lipid complex, can be found in both cells with impaired autophagy and senescent cells. As shown in Figure 4A,B, the changes in the protein level of LC3BII showed that autophagy was initiated. However, we also observed that autophagic flux was dramatically decreased, accompanied by the accumulation of lipofuscin, suggesting that autophagy was impaired by diTFPP/C_2_-ceramide cotreatment (Figure 4C). Furthermore, our results showed that diTFPP/C_2_-ceramide cotreatment decreased the number of SA-gal-positive cells, a hallmark of senescence (Figure 4D), suggesting that lipofuscin accumulation in these cells may not be due to the senescence mechanism. Senescent cells have been shown to be insensitive to both extrinsic and intrinsic stimuli, such as apoptosis induction, suggesting that cancer cells may escape cell death by acquiring a reversible senescence-like phenotype (SLP). For example, senescent cells with low protein levels of the antiapoptotic protein Bcl-2 are resistant to H_2_O_2_-induced cell death [62]. Similarly, our previous study suggested that SLP may endow breast cancer cells with resistance to C_2_-ceramide treatment [19].

### 3.5. diTFPP-Induced LAMP2 Modification Sensitizes HCC Cells to Autophagic Stress

Glycosylation is one of the most important modifications on the lysosomal membrane, and various highly glycosylated proteins, including lysosome-associated membrane protein 1 (LAMP1) and LAMP2, can integrate into the lysosomal membrane. These fully glycosylated LAMP2 proteins are considered to protect the lysosomal membrane from enzyme-mediated degradation [63]. However, improper modification, such as insufficient glycosylation (a complete lack of glycosylation or hypoglycosylation) of LAMP2, not only fails to protect the lysosomal membrane from self-digestion by lysosomal enzymes but also affects autophagosome–lysosome fusion during autophagy. During autophagy, cytoplasmic GRASP55 functions as a bridge between LC3-II (on autophagosomes) and LAMP2 by interacting with LC3-II and LAMP2, facilitating autophagosome–lysosome fusion [64].

**Figure 5:**
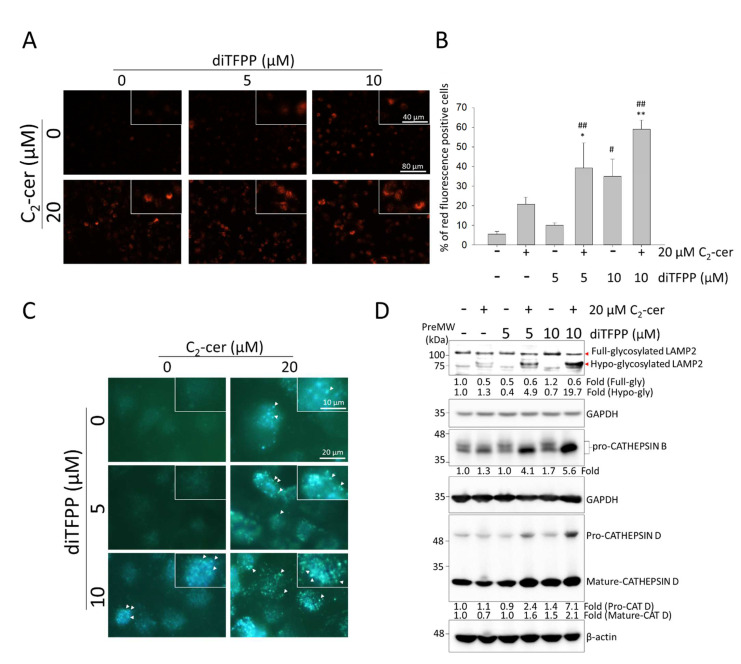

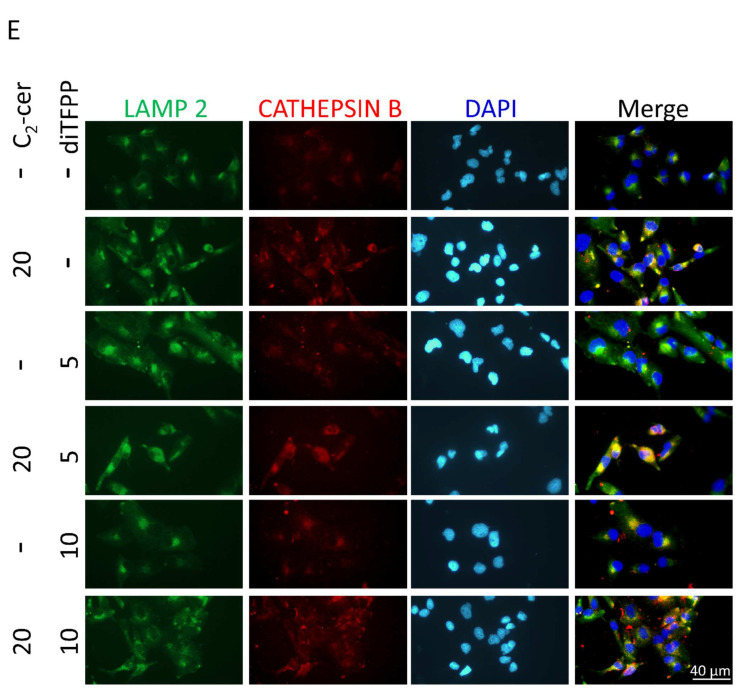
Changes in lysosomal pH during autophagy following diTFPP/C_2_-ceramide cotreatment for 24 h. (**A**) Accumulation of lysosomes was detected using LysoTracker fluorescence staining and observed by fluorescence microscopy. (**B**) Quantitative analysis of acidic organelle accumulation using flow cytometry-based LysoTracker fluorescence staining. Red fluorescence indicates lysosomes # *p* < 0.05 and ## *p* < 0.01, * *p* < 0.05 and ** *p* < 0.01. (**C**) Changes in lysosomal pH were evaluated by staining with a pH-sensitive LysoSensor™ probe. White arrows indicate the fluorescent puncta where the acidic organelles, especially the lysosomes, localize. (**D**) Changes in the levels of lysosome-associated proteins were evaluated using Western blot analysis. (**E**) The subcellular localization and fluorescence intensity of LAMP2 and CATHEPSIN B were evaluated using an immunofluorescence assay. PreMW: prestained protein molecular weight marker (see Section 2.4).

As shown in Figure 5A,B, diTFPP treatment caused the accumulation of lysosomes (acidic organelles), and staining with a pH-sensitive LysoSensor, a ratiometric probe used to assess changes in lysosomal pH, further confirmed the increase in mature lysosomes (Figure 5C).

Previous studies indicated that LAMP2 hypoglycosylation may be due to defects in the Conserved Oligomeric Golgi (COG) complex (known as COG-Congenital Disorders of Glycosylation (COG-CDG)) or because the key enzymes α-1,3-mannosyl-glycoprotein 2-β-N -acetylglucosaminyltransferase (MGAT1, which regulates N-terminal glycosylation) and UDP-glucose 4-epimerase (GALE, which regulates O-glycosylation) are inactive [65]. N-glycosylation contributes more to LAMP2 mobility (referred to as a shift on SDS PAGE) than O-glycosylation, and O-glycosylation contributes to LAMP2 protein stability [65].

Our study showed that C_2_-ceramide treatment caused moderate hypoglycosylation of LAMP2 (with a lower molecular weight), while the presence of diTFPP significantly increased LAMP2 hypoglycosylation without significantly affecting the protein level of LAMP2 (Figure 5D). Therefore, our results suggested that the N-glycosylation of LAMP2 may be changed instead of O-glycosylation, which may explain the shifting of LAMP2 to a lower molecular weight compared with the other treated groups (Figure 5D).

Similarly, upregulation of the lysosomal proteases CATHEPSIN-B and CATHEPSIN-D was detected after cotreatment with C_2_-ceramide and diTFPP (Figure 5D). Furthermore, immunofluorescence staining confirmed the increase in CATHEPSIN B (Figure 5E). On the basis of the above observations, we suggest that C_2_-ceramide/diTFPP cotreatment results in an increase in LAMP2 hypoglycosylation, which may impair autophagosome–lysosome fusion and result in the dramatic accumulation of lysosomes, ultimately sensitizing HCC cells to cell death. Our study showed that C_2_-ceramide treatment caused moderate hypoglycosylation of LAMP2 (with a lower molecular weight), while the presence of diTFPP significantly increased LAMP2 hypoglycosylation (Figure 5D). Similarly, upregulation of the lysosomal proteases CATHEPSIN-B and -D was detected after cotreatment with C_2_-ceramide and diTFPP (Figure 5D). Furthermore, immunofluorescence staining confirmed the increase in CATHEPSIN B (Figure 5E). On the basis of the above observations, we suggest that C_2_-ceramide/diTFPP cotreatment results in an increase in LAMP2 hypoglycosylation, which may impair autophagosome–lysosome fusion and result in dramatic accumulation of lysosomes, ultimately sensitizing HCC cells to cell death.

### 3.6. diTFPP/C_2_-Ceramide Treatment Enhances ROS Production and ER Stress to Cause Autophagic Stress

**Figure 6:**
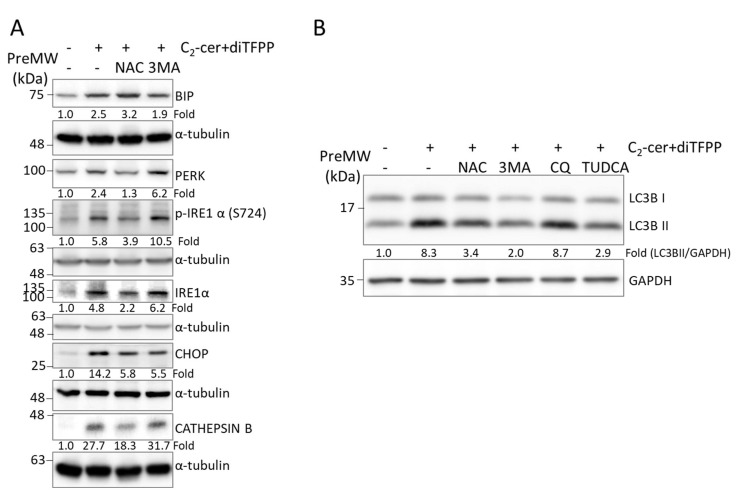
Roles of ER stress and autophagy activation in HCC cells following C_2_-ceramide/diTFPP cotreatment. To validate the roles of ROS production, ER stress and autophagy initiated in HCC cells cotreated with ceramide and diTFPP, HCC cells were pretreated with the ROS scavenger NAC (10 mM), autophagy inhibitors 3-MA (2 mM) and CQ (20 μM), and ER stress inhibitor TUDCA (100 μM) for 6 h and with 10 μM diTFPP and 20 μM C2-ceramide for 24 h. (**A**) Assessment of ER stress-associated proteins and CATHEPSIN B. (**B**) Assessment of the autophagy indicator LC3B II. α-Tubulin and GAPDH were used as the internal controls. PreMW: prestained protein marker (see Section 2.4).

As shown in Figure 4A, diTFPP/C_2_-ceramide cotreatment led to a reduction in autophagic flux and attenuation of autophagic degradation (Figure 4B), suggesting that autophagic stress was increased. Prior to treatment with diTFPP/C_2_-ceramide, we treated HCC cells with the ROS scavenger N-acetylcysteine (NAC); two autophagy inhibitors, 3-methyladenine (3-MA) and CQ; and the ER stress inhibitor tauroursodeoxycholic acid (TUDCA). The Western blot analysis results showed that the levels of the ER stress-related protein CATHEPSIN B and the autophagic stress-related protein LC3BII in diTFPP/C_2_-ceramide-cotreated HCC cells were reduced, suggesting that ROS scavenging alleviates the ER stress and autophagic stress caused by diTFPP/C_2_-ceramide-induced ROS (Figure 6). In contrast, our results showed that blocking ER stress ameliorates the accumulation of LC3BII, suggesting that the increase in endogenous ROS induces ER stress and, eventually, autophagic stress. In addition, we used specific inhibitors to examine whether LAMP2 modifications affect the ROS/ER stress/autophagic stress axis; however, none of the tested inhibitors rescued the hypoglycosylation (lower molecular mass) of LAMP2 in HCC cells following diTFPP/C_2_-ceramide treatment (Figure S2), suggesting that LAMP2 glycosylation may be regulated by other mechanisms.

## 4. Conclusions

**Figure 7:**
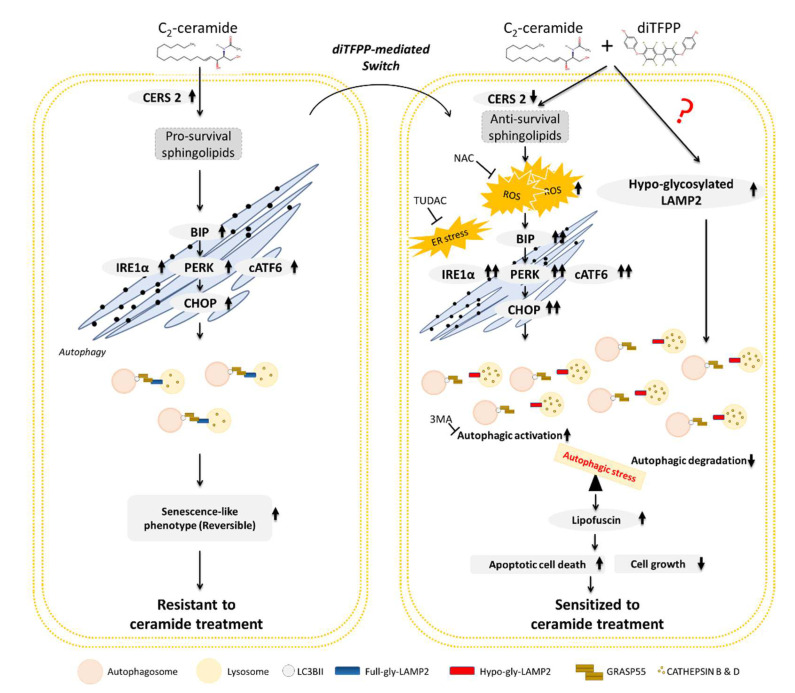
Proposed role of diTFPP in C_2_-ceramide-induced death and growth arrest in HCC cells. The metabolism of endogenous sphingolipids favors the conversion of C_2_-ceramide to a prosurvival sphingolipid, inducing mild ER stress and autophagy. Reversible SLP may attenuate C_2_-ceramide-induced anti-HCC effects, such as proliferation inhibition and apoptosis induction, rendering HCC cells resistant to C_2_-ceramide treatment. However, diTFPP promotes the conversion of prosurvival sphingolipids to proapoptotic sphingolipids and increases C_2_-ceramide-induced cellular stresses, including oxidative stress, ER stress, and autophagic stress. In contrast, C_2_-ceramide/diTFPP cotreatment also decreases the full glycosylation of LAMP2, a hallmark of mature lysosomes, impairing autophagosome–lysosome fusion and contributing to the severity of autophagic stress. Thus, the abovementioned stress sensitizes HCC cells to C_2_-ceramide-induced apoptosis. Full-gly LAMP2: fully glycosylated LAMP2; hypo-gly LAMP2: hypoglycosylated LAMP2.

In conclusion, treatment with C_2_-ceramide alone increases the protein level of CERS2, modulating sphingolipid metabolism to favor the conversion of C_2_-ceramide to a prosurvival sphingolipid and activating the UPR, including the PERK, IRE1α, and ATF6 pathways, to alleviate misfolded protein-induced stress in HCC cells. UPR activation may further initiate autophagy and SLP, ultimately contributing to the resistance of HCC cells to C_2_-ceramide treatment. In the presence of diTFPP, the protein level of CERS2 is downregulated, and ceramide metabolism favors its conversion to antisurvival sphingolipids. Additionally, the presence of diTFPP increases oxidative stress, causing massive accumulation of un- or misfolded proteins to activate the UPR to ER stress, resulting in increased initiation of autophagy to clear the accumulated misfolded proteins. However, diTFPP/C_2_-ceramide cotreatment results in insufficient glycosylation of LAMP2 on lysosomes, which in turn attenuates or blocks autophagosome–lysosome fusion, a critical process in autophagy. Autophagic stress and the accumulation of numerous metabolites and complexes of misfolded proteins lower the threshold for triggering cell death in response to C_2_-ceramide. Eventually, diTFPP sensitizes HCC cells to C_2_-ceramide-induced antiproliferation and apoptosis (Figure 7).

## Data Availability

The authors confirm that the data supporting the findings of this study are available within the article and will be provided on request.

## Supplementary Materials

The following data are available online at https://www.mdpi.com/article/10.3390/cancers14102528/s1, Figure S1: Effect of diTFPP on the level of the sphingolipid metabolic enzyme CERS2. C_2_-ceramide treatment alone upregulated the prosurvival sphingolipid metabolic enzyme CERS2, whereas diTFPP/C_2_-ceramide cotreatment dramatically decreased the protein level of CERS2. β-Actin was used as the internal control. PreMW: prestained protein molecular weight marker (see Section 2.4). Figure S2: Effect of ROS scavengers, autophagy and ER stress inhibitors on HCC cells treated with ceramide and diTFPP. HCC cells were pretreated with the ROS scavenger NAC, autophagy inhibitors 3-MA and CQ, and ER stress inhibitor TUDCA for 6 h. The protein level of LAMP2 was measured after cells were treated with diTFPP/C_2_-ceramide for 24 h. α-Tubulin was used as the internal control. The bands with a high molecular mass (above 75 to 100 kDa) indicate glycosylated LAMP2. The band with a low molecular mass (approximately 75 kDa) indicates un or hypoglycosylated LAMP2. α-Tubulin was used as the internal control. PreMW: prestained protein molecular weight marker (see Section 2.4); File S1: Original WB.
