# Supplementary material for: diTFPP, a Phenoxyphenol, Sensitizes Hepatocellular Carcinoma Cells to C2-Ceramide-Induced Autophagic Stress by Increasing Oxidative Stress and ER Stress Accompanied by LAMP2 Hypoglycosylation
Source: Cancers (Basel). 2022 May 20;14(10):2528. doi: 10.3390/cancers14102528 (PMC9139631; doi:10.3390/cancers14102528)
Supplement: Supplementary file 1 [file cancers-14-02528-s001.zip › cancers-1726601-Figures S1 and S2.pdf]

Figure S1

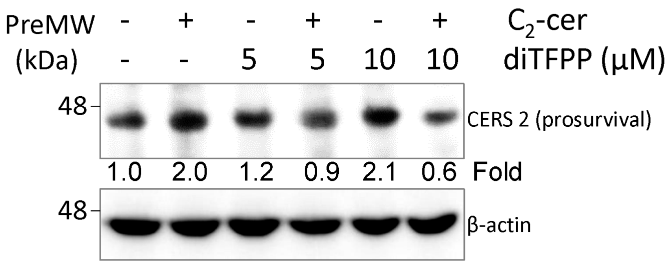

Figure S1. Effect of diTFPP on the level of the sphingolipid metabolic enzyme CERS2. C_2-ceramide treatment alone upregulated the prosurvival sphingolipid metabolic enzyme CERS2, whereas diTFPP/C_2-ceramide cotreatment dramatically decreased the protein level of CERS2. β-Actin was used as the internal control. PreMW: prestained protein molecular weight marker (see Section 2.4).

Figure S2

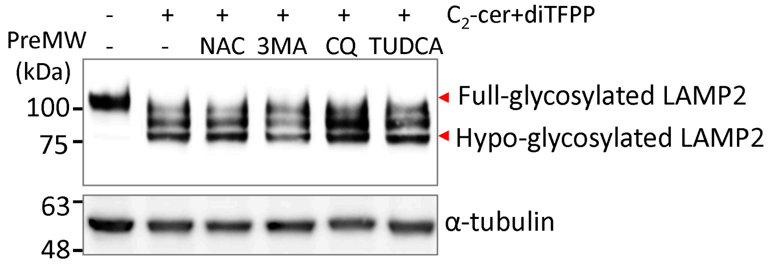

Figure S2. Effect of ROS scavengers, autophagy and ER stress inhibitors on HCC cells treated with ceramide and diTFPP. HCC cells were pretreated with the ROS scavenger NAC, autophagy inhibitors 3-MA and CQ, and ER stress inhibitor TUDCA for 6 h. The protein level of LAMP2 was measured after cells were treated with diTFPP/C_2-ceramide for 24 h. α-Tubulin was used as the internal control. The bands with a high molecular mass (above 75 to 100 kDa) indicate glycosylated LAMP2. The band with a low molecular mass (approximately 75 kDa) indicates un or hypoglycosylated LAMP2. α-Tubulin was used as the internal control. PreMW: prestained protein molecular weight marker (see Section 2.4).
