# Supplementary material for: diTFPP, a Phenoxyphenol, Sensitizes Hepatocellular Carcinoma Cells to C2-Ceramide-Induced Autophagic Stress by Increasing Oxidative Stress and ER Stress Accompanied by LAMP2 Hypoglycosylation
Source: Cancers (Basel). 2022 May 20;14(10):2528. doi: 10.3390/cancers14102528 (PMC9139631; doi:10.3390/cancers14102528)
Supplement: Supplementary file 1 [file cancers-14-02528-s001.zip › cancers-1726601-File S1.pdf]

Figure S1

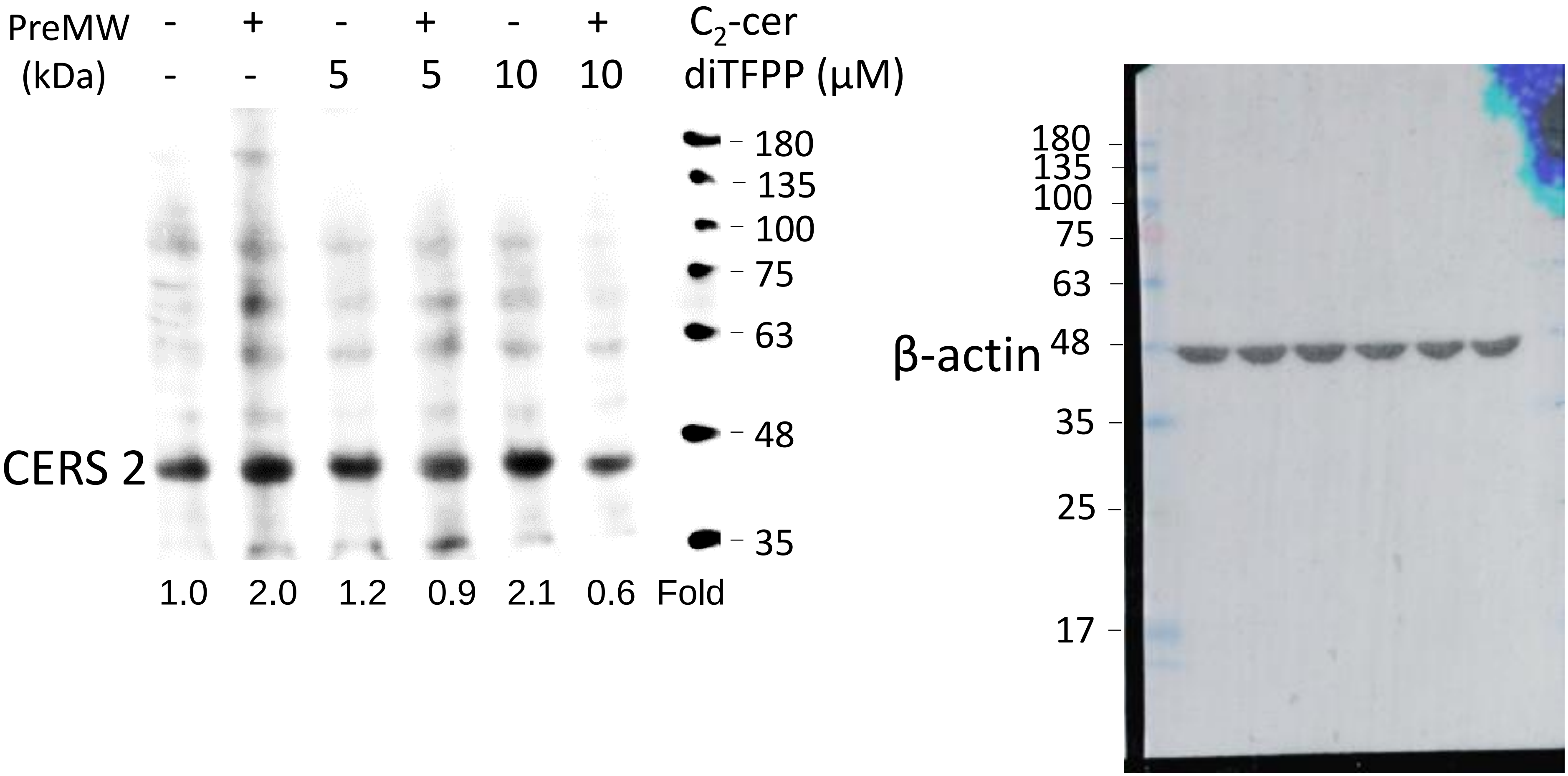
PreMW (kDa)
C2-cer: - + - + - +
diTFPP (μM): - - 5 5 10 10
180
135
100
75
63
48
35
CERS 2
1.0 2.0 1.2 0.9 2.1 0.6 Fold
β-actin
180
135
100
75
63
48
35
25
17

Figure 3

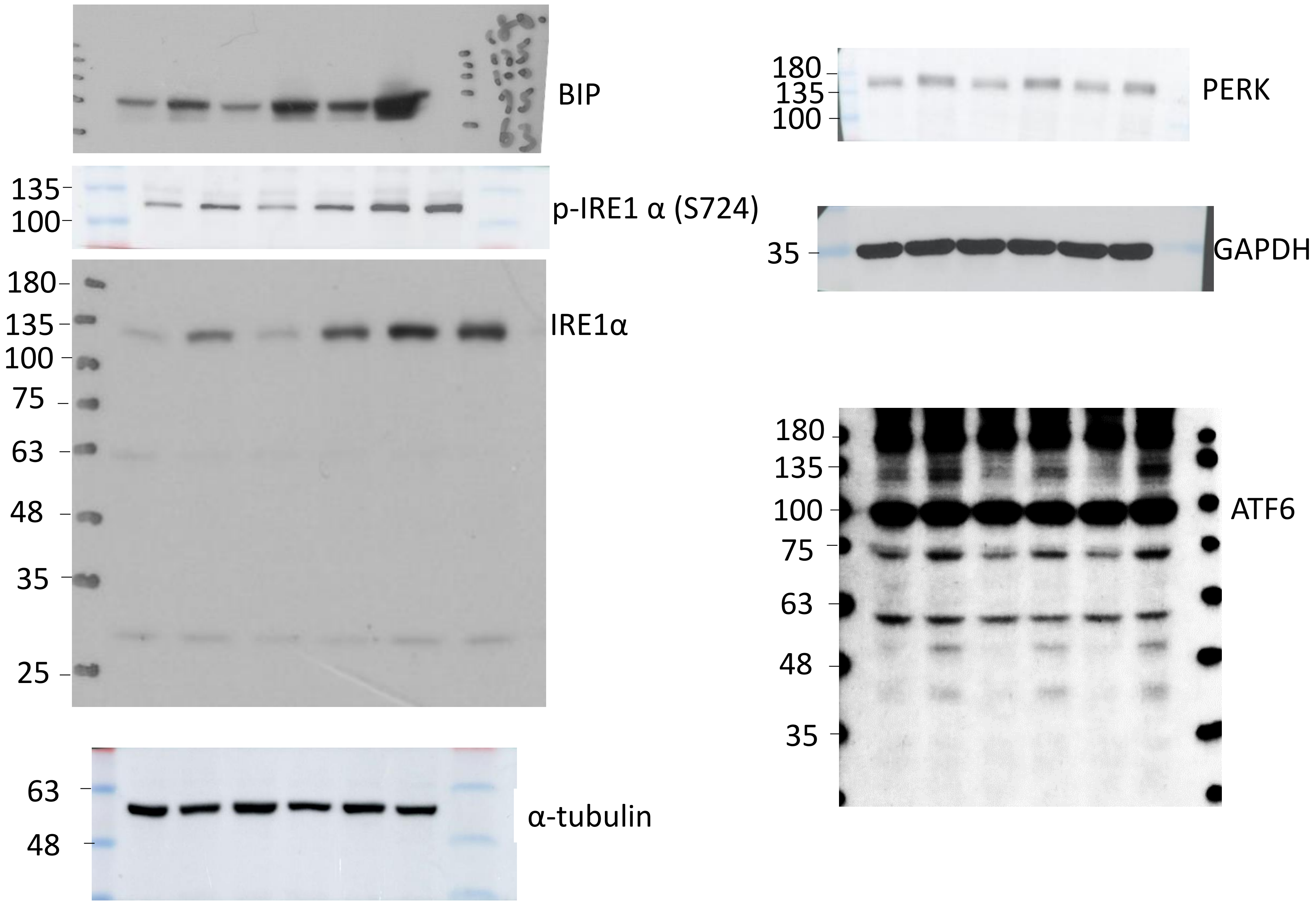
BIP
180
135
100
95
63
135
100
p-IRE1 α (S724)
180
135
100
75
63
48
35
25
IRE1α
63
48
α-tubulin
180
135
100
PERK
35
GAPDH
180
135
100
75
63
48
35
ATF6

Figure 3-continue

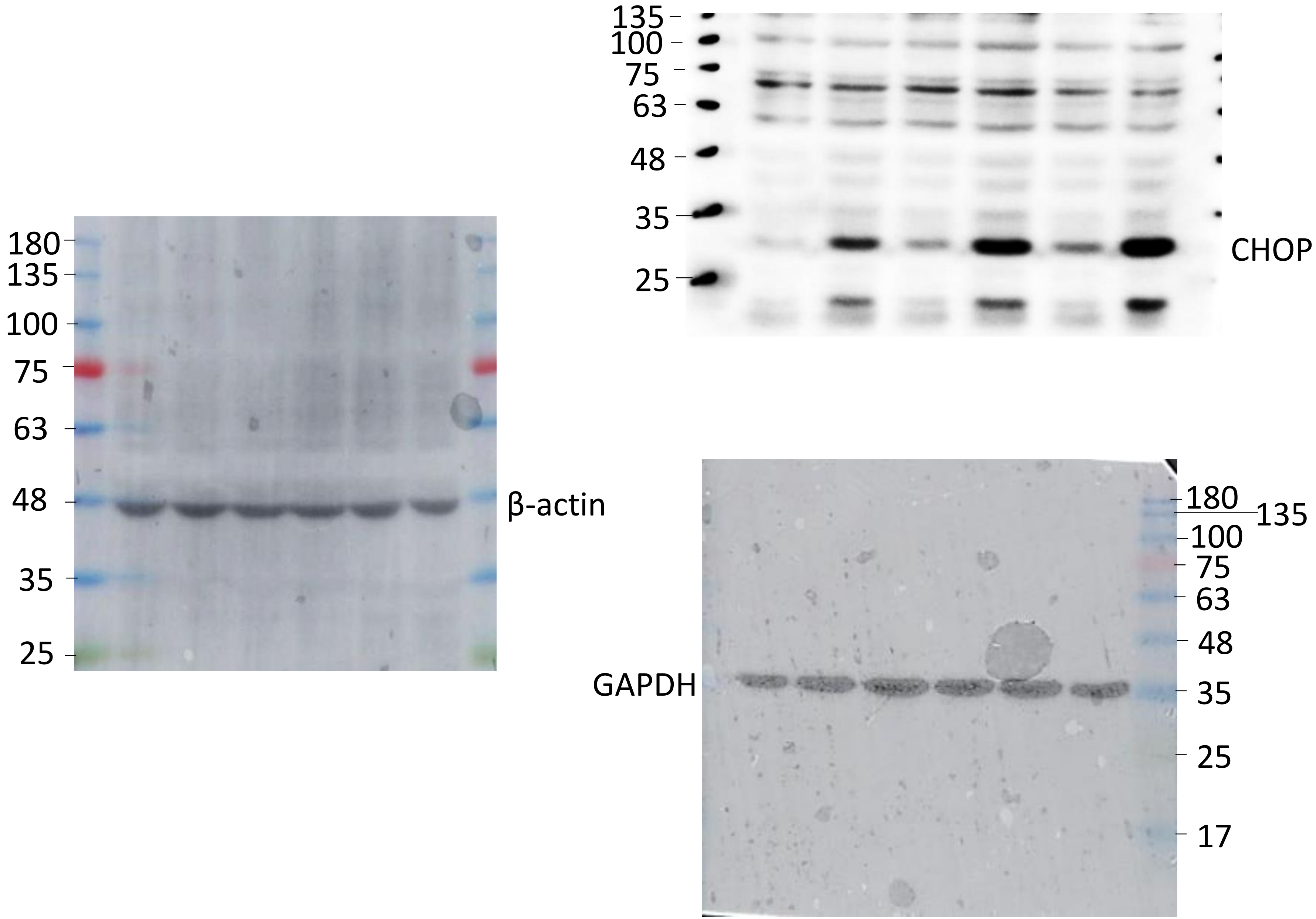

Figure 4

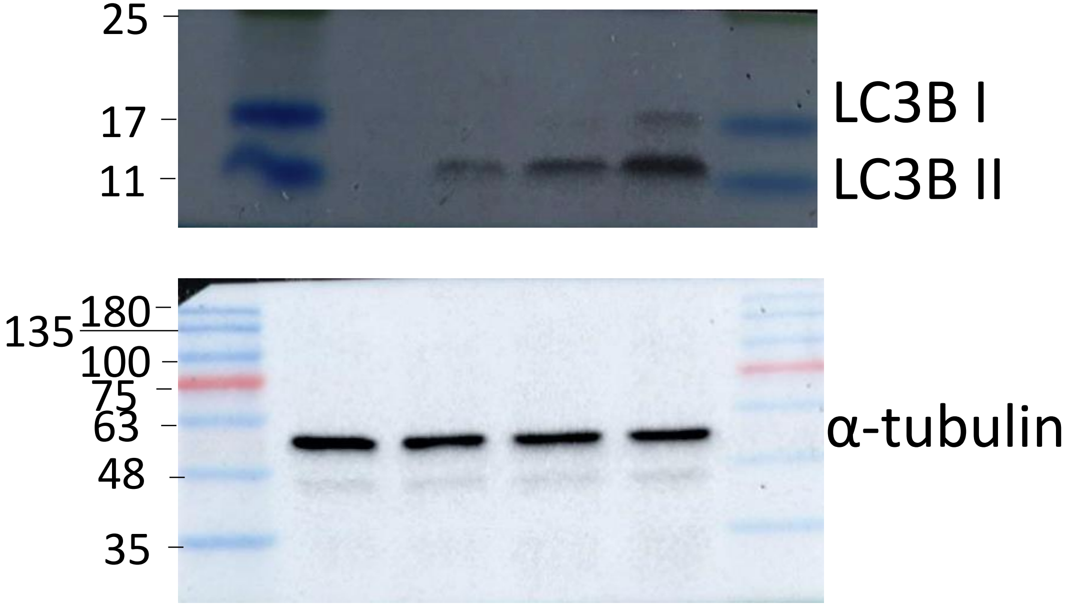

Figure 5

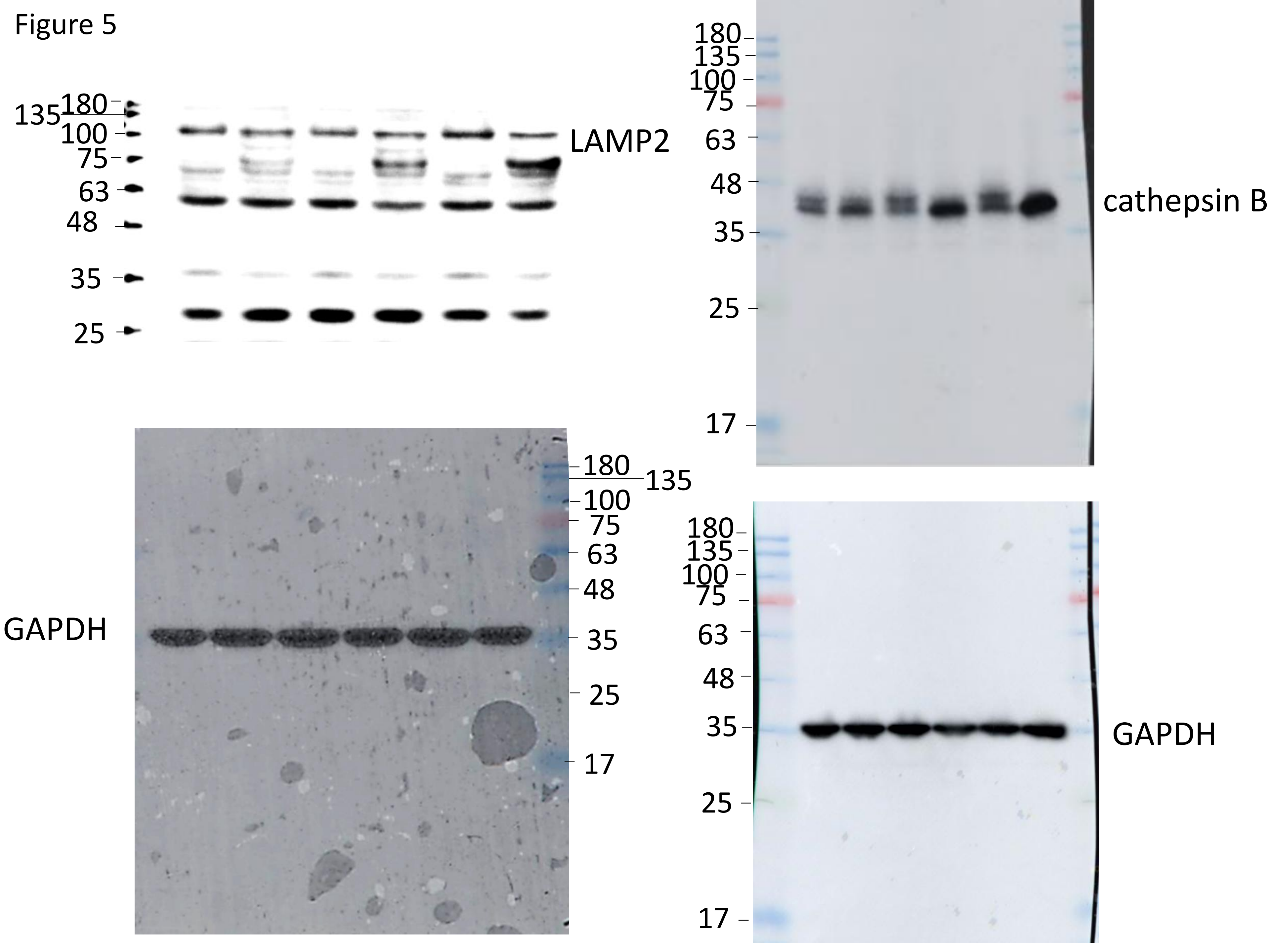
LAMP2
180
135
100
75
63
48
35
25
cathepsin B
180
135
100
75
63
48
35
25
17
GAPDH
180
135
100
75
63
48
35
25
17
GAPDH
180
135
100
75
63
48
35
25
17

Figure 5-continue

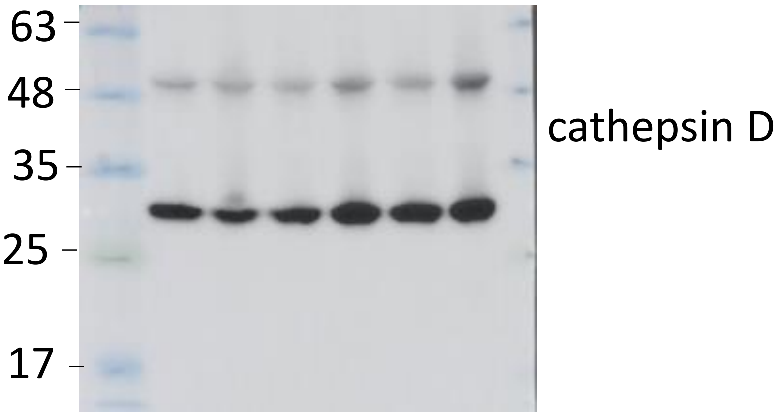

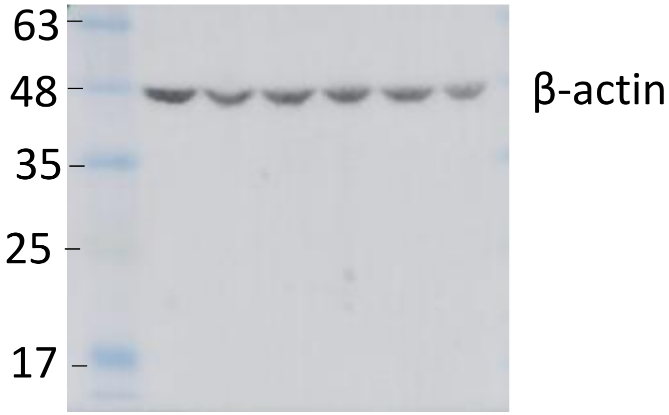

Figure 6A

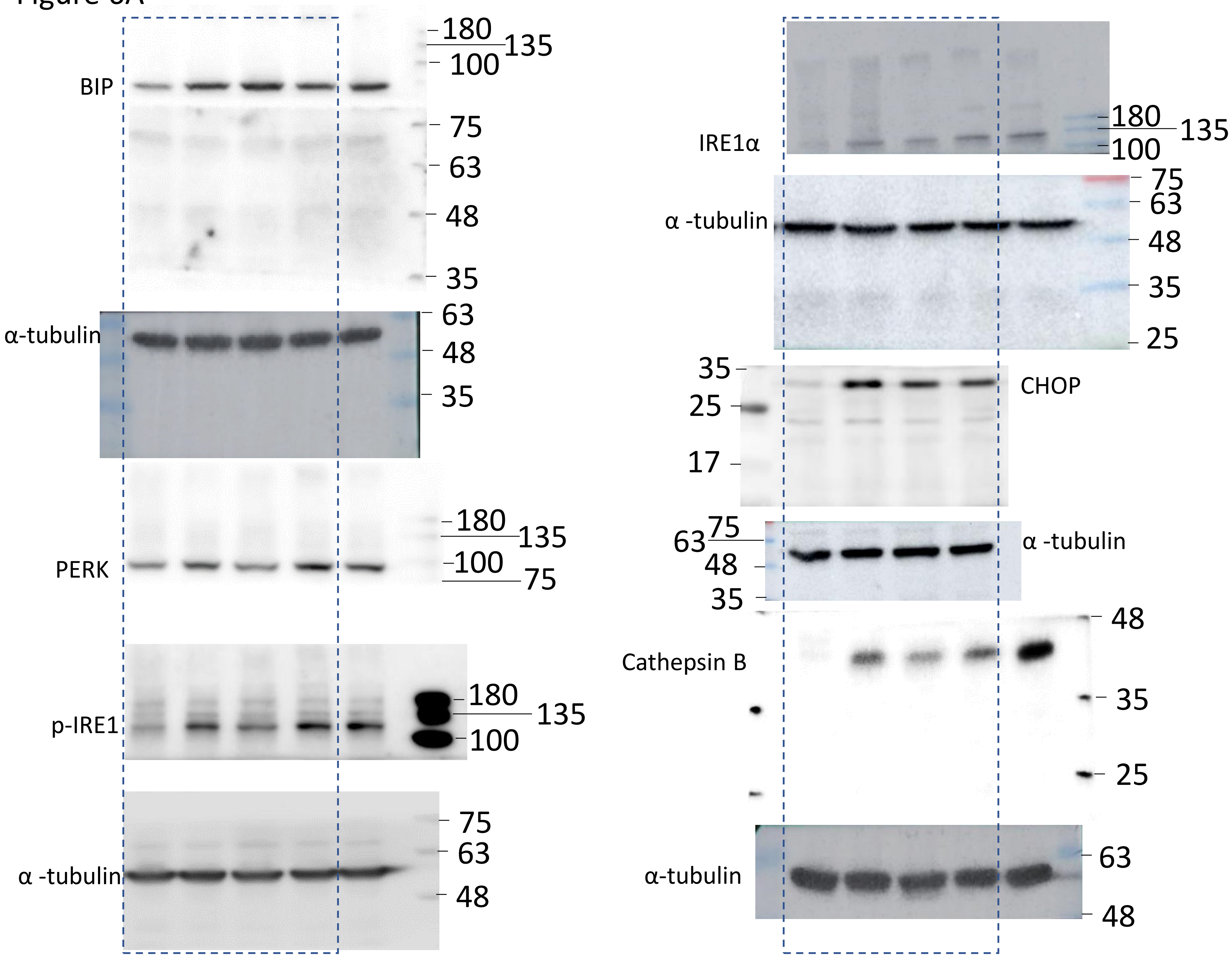

BIP
180
135
100
75
63
48
35
α-tubulin
63
48
35
PERK
180
135
100
75
p-IRE1
180
135
100
α -tubulin
75
63
48
IRE1α
180
135
100
α -tubulin
75
63
48
35
25
CHOP
35
25
17
α -tubulin
75
63
48
35
Cathepsin B
48
35
25
α-tubulin
63
48

Figure 6B

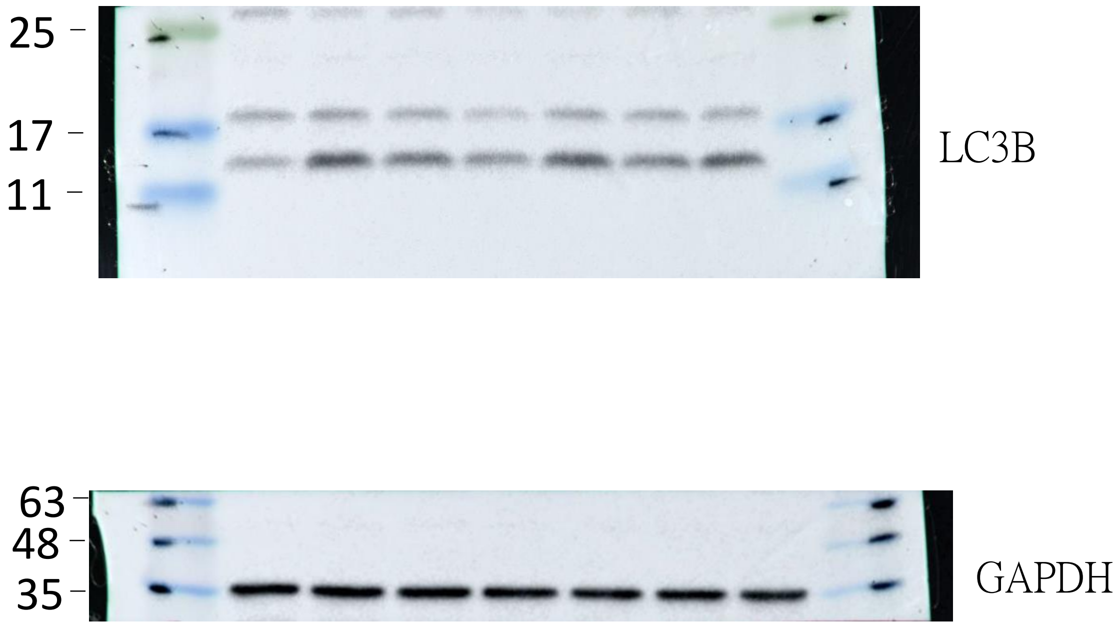

Figure S2

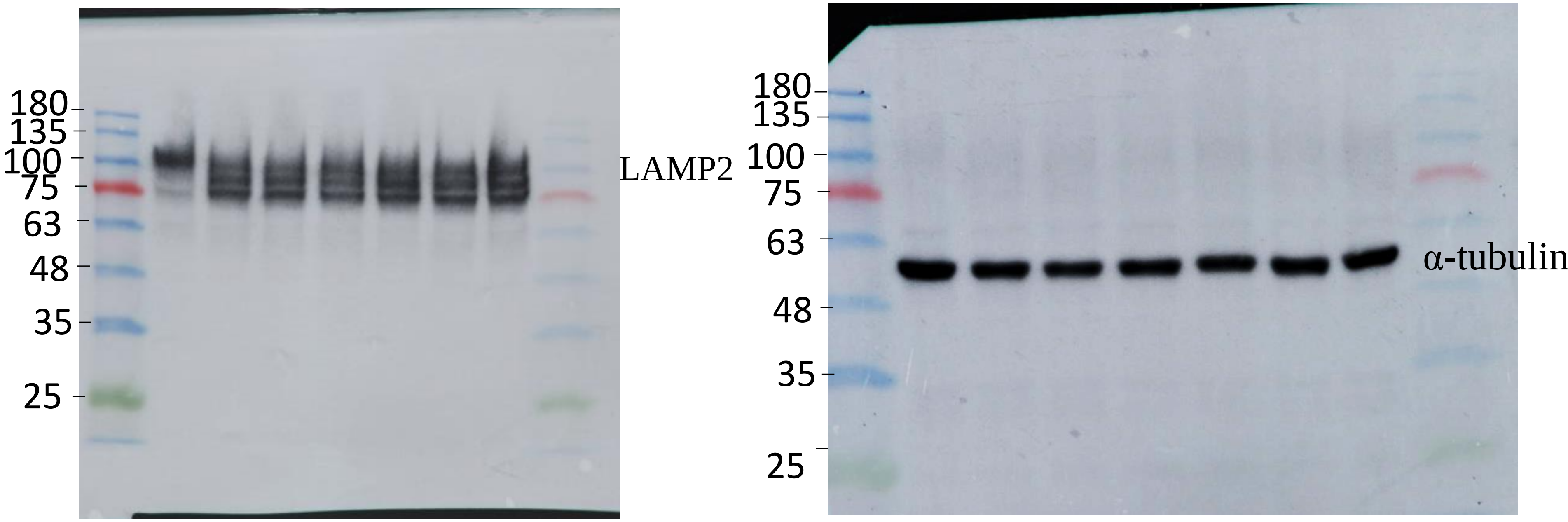
180
135
100
75
63
48
35
25
LAMP2
180
135
100
75
63
48
35
25
α-tubulin
